# The Use of Stem Cell-Derived Organoids in Disease Modeling: An Update

**DOI:** 10.3390/ijms22147667

**Published:** 2021-07-17

**Authors:** Joseph Azar, Hisham F. Bahmad, Darine Daher, Maya M. Moubarak, Ola Hadadeh, Alissar Monzer, Samar Al Bitar, Mohamed Jamal, Mohamed Al-Sayegh, Wassim Abou-Kheir

**Affiliations:** 1 Department of Anatomy, Cell Biology and Physiological Sciences, Faculty of Medicine, American University of Beirut, Beirut 1107 2260, Lebanon; jha11@mail.aub.edu (J.A.); hfbahmad@gmail.com (H.F.B.); dkd04@mail.aub.edu (D.D.); mmm122@mail.aub.edu (M.M.M.); hadadeh.ola@gmail.com (O.H.); aam48@mail.aub.edu (A.M.); sfa28@mail.aub.edu (S.A.B.); 2 Arkadi M. Rywlin M.D. Department of Pathology and Laboratory Medicine, Mount Sinai Medical Center, Miami Beach, FL 33140, USA; 3 Hamdan Bin Mohammed College of Dental Medicine, Mohammed Bin Rashid University of Medicine and Health Sciences, Dubai 66566, United Arab Emirates; 4 Biology Division, New York University Abu Dhabi, Abu Dhabi 2460, United Arab Emirates

**Keywords:** pluripotent stem cells, embryonic stem cells, organoids, disease modeling, 3D culturing

## Abstract

Organoids represent one of the most important advancements in the field of stem cells during the past decade. They are three-dimensional in vitro culturing models that originate from self-organizing stem cells and can mimic the in vivo structural and functional specificities of body organs. Organoids have been established from multiple adult tissues as well as pluripotent stem cells and have recently become a powerful tool for studying development and diseases in vitro, drug screening, and host–microbe interaction. The use of stem cells—that have self-renewal capacity to proliferate and differentiate into specialized cell types—for organoids culturing represents a major advancement in biomedical research. Indeed, this new technology has a great potential to be used in a multitude of fields, including cancer research, hereditary and infectious diseases. Nevertheless, organoid culturing is still rife with many challenges, not limited to being costly and time consuming, having variable rates of efficiency in generation and maintenance, genetic stability, and clinical applications. In this review, we aim to provide a synopsis of pluripotent stem cell-derived organoids and their use for disease modeling and other clinical applications.

## 1. Introduction

**Figure 1:**
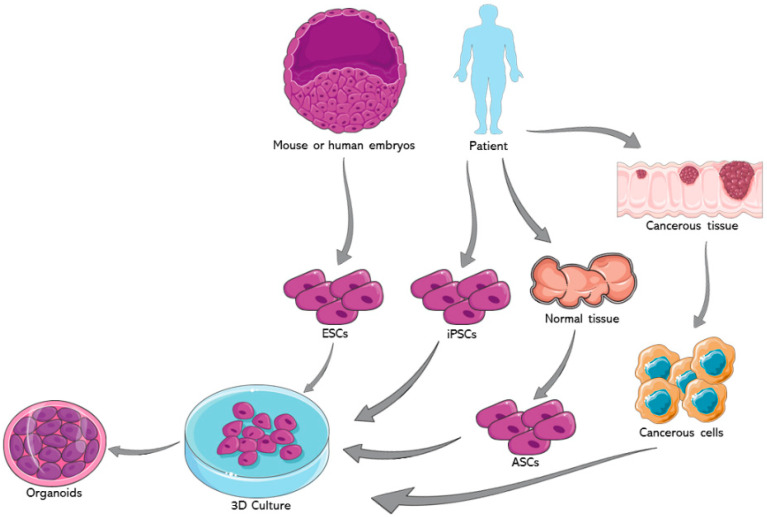
Organoids generation from different types of stem cells. Organoids can be generated from induced pluripotent stem cells (iPSCs), embryonic stem cells (ESCs) and patient-derived adult stem cells from normal and cancerous tissues (ASCs). Cells are usually grown in three dimensional systems under specific conditions to stimulate the growth of organoids. Shapes and images are imported from Servier Medical Art by Servier (http://smart.servier.com/), accessed on 24 May 2021. Licensed under a Creative Commons Attribution 3.0 Unported License (https://creativecommons.org/licenses/by/3.0/).

Organoids represent one of the most important advancements in the field of stem cells during the past decade. The earliest usage of the term goes back to 1946 when Smith and Cochrane described a cystic teratoma case by referring to it as “cystic organoid teratoma” [1]. Organoids, or as the term literally signifies “resembling an organ”, are three-dimensional (3D) in vitro culturing systems that originate from self-organizing stem cells, capable of mimicking the in vivo structural and functional specificities of an organ [2]. They can be derived from either pluripotent stem cells (PSCs) or organ-specific adult stem cells (ASCs) [3]. PSCs, for instance, are capable of generating in vitro tissue models recapitulating what happens in in vivo organogenesis, where tissues are derived from embryonic stem cells (ESCs) [4]. Figure 1 summarizes the potential sources for organoids development.

Organoids have a great potential to be used in a multitude of fields [5,6]. One such avenue is cancer research, particularly through the development of tumor organoids. For example, glioblastoma organoids were established with the hopes of creating a satisfactory in vitro model that reflects the in vivo hypoxic gradients and heterogeneity of this greatly heterogenous cancer. This glioblastoma model can be used for both diagnosis and therapeutic purposes [7]. Furthermore, our group succeeded in generating and characterizing patient-derived prostate organoids from fresh primary tissue specimens. We confirmed the validity of those organoids as a relevant in vitro model to assess personalized treatment responses wherein we observed a differential drug response between different patient samples, at the protein and gene levels [8]. Moreover, we were able to isolate novel patient-derived prostate epithelial cells from the generated organoids [8]. Organoid models were also used in cancer immunotherapy research. In fact, Jenkins et al. used patient and murine derived organoids to establish an ex vivo profiling of PD-1 blockade. This would significantly help in dissecting the tumor microenvironment, enhancing precision immune-oncologic therapies, and ultimately deciphering the mechanisms behind immune checkpoint blockade resistance [9]. Furthermore, Dijkstra et al. co-cultured epithelial tumor organoids with peripheral blood lymphocytes to expand the population of tumor-reactive T-cells. This co-culture model can be also employed to characterize the sensitivity of neoplastic cells to the T cell-mediated killing at a personalized patient level [10]. Another potential avenue for the usage of organoids is hereditary diseases such as cystic fibrosis (CF), which maintain the tissue of origin’s genetic fingerprint. Beekman’s group was able to elucidate a quantifying assay of the CF transmembrane conductance regulator (CFTR) function, the malfunctioning chloride channel in CF, using an intestinal model that closely mimics the core aspects of the disease’s in vivo characteristics. This can aid in diagnosis and potentially provides novel therapeutic approaches to enhance drug development strategies for the disease [11]. After the COVID-19 pandemic has swept the world, organoids are proving to be of increasing value as viral replication platforms that can help expand our understanding of this virus [12].

**Figure 2:**
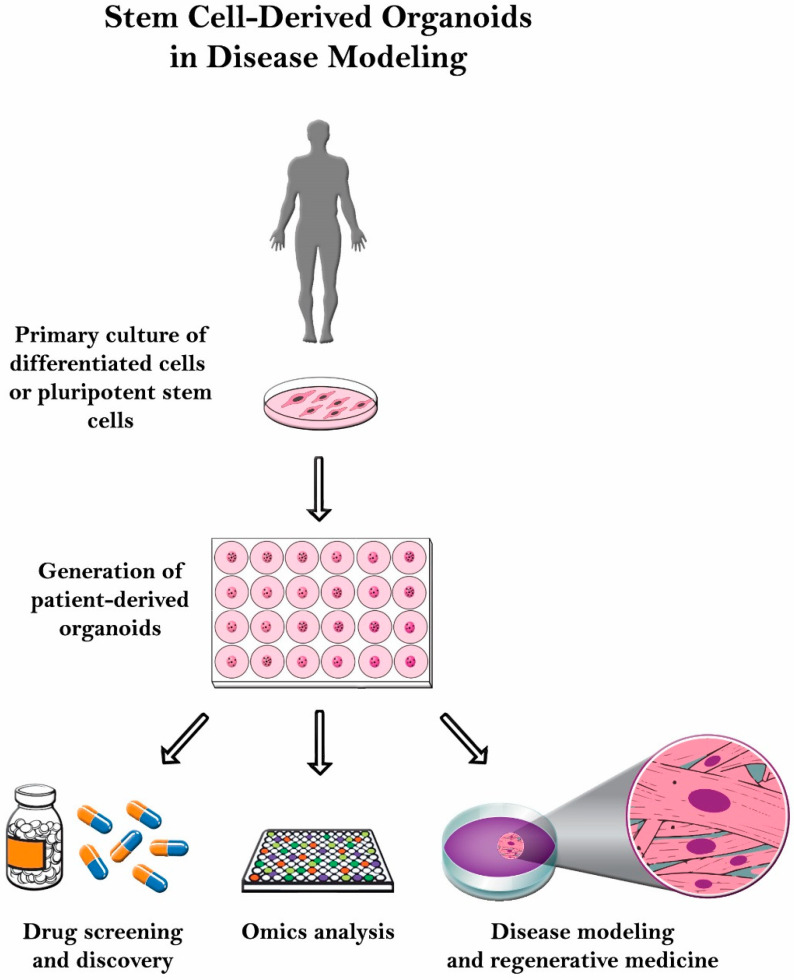
Schematic illustrating the use of stem cell-derived organoids for various biomedical and clinical applications including drug screening and discovery, disease modeling, genetic manipulation, omics analysis, and regenerative medicine among many others.

Even though it has significant potential, organoid culturing is still rife with many challenges. Despite these systems’ heterogeneity, the tumor microenvironment is often not properly reconstituted since most of the patient-derived organoids (PDOs) do not have the supporting stroma [13]. Other challenges observed with glioblastoma patient-derived xenotransplantation (PDX) models include cost, time consumption, and variable rates of transplantation [14]. Another limitation with the PDX system is that the immune response cannot be assessed properly since the host strains used are immunodeficient. Therefore, there is a poor assessment on how the immune system would have responded [15]. In this review, organoid culture will be thoroughly discussed along with its various applications in disease modeling (Figure 2).

## 2. Modeling Diseases Using Induced Pluripotent Stem Cells (Ipscs)-Derived Organoids

Over the years, major advances and discoveries in stem cell research have been made in PSCs. PSCs are characterized by their self-renewal capacity and potency to differentiate into specialized cell types deriving from the three germ layers such as ectoderm, endoderm, or mesoderm [16]. Stem cells in general are divided into five basic categories including ESCs, very small embryonic-like stem cells, nuclear transfer stem cells, ASCs, and reprogrammed stem cells which include iPSCs [17].

### 2.1. Introduction to iPSC Technology

Human-induced pluripotent stem cells (hiPSCs) are often isolated from somatic cells such as fibroblasts and reprogrammed to an embryonic-like state by virally adding pluripotency-inducing transcription factors Oct3/4, Sox2, Klf4, and c-Myc [18]. However, to make iPSCs clinically applicable, non-integrating approaches including episomal DNAs [19], adenovirus [20], Sendai virus [21], PiggyBac transposons [22], minicircles [23], recombinant proteins [24], synthetically modified mRNAs [25], microRNAs [26], and small molecules [27], were used to avoid insertional mutagenesis and genetic alterations associated with the retroviral and lentiviral mediated introduction of reprogramming factors [28].

Human iPSC technology has advanced rapidly and been employed in disease modeling, regenerative medicine, gene editing, drug discovery, and pharmacology [29,30,31]. Given their human origin in which drugs are tested and transplantations are received, iPSCs are widely used due to their accessibility, abundant reproducibility, potency to differentiate into all cell types, application in personalized medicine and avoidance of the ethical concerns associated with human ESCs [32]. Moreover, they provide high-throughput screening frameworks to distinguish novel compounds as potential leads to treat human diseases [33,34]. In addition, hiPSCs are distinctive in their ability to differentiate into specific human cell types of interest which allows for the selection of the tested compounds and cell-based screening to identify any off-targets [35].

The rapid generation of genetically established human iPSC-based disease models has expanded thanks to advances in gene editing technologies, especially the CRISPR–Cas9 technology. Combining iPSC and CRISPR/Cas9 technologies facilitated the investigation of the molecular and cellular mechanisms underlying inherited disease [36]. iPSCs are an essential source to develop 3D-architectured cellular networks such as organoids which recapitulate organ architecture and physiology [37,38]. As a result, human iPSC technology holds a lot of promise for human disease modeling, drug development, and stem cell-based therapy. Here we will review the main applications of iPSCs technology in terms of disease modeling, drug screening and therapeutics.

### 2.2. iPSC-Derived Models in Biomedical Application: Complex Tissues in a Dish

Organoids could be derived from PSCs; which are either ESCs or iPSCs, and tissue-specific resident stem cells such as organ-specific ASCs [2]. Organoids are distinct in that they are self-organizing 3D culture structures that highly mimic the native human organs [39]. Organoid generation revolves around three main steps. First, is the induction or inhibition of the key signaling pathways to develop the appropriate regional identity during stem cell differentiation. Second, is generating media formulations that allow adequate terminal differentiation of the needed cell types within the organoid, based on established 2D culture methods or murine developmental process. Finally, cells are grown to propagate in three dimensions either by combining cells into 3D structures or by implementing them into a 3D matrix [39]. Organoid differentiation from PSCs has gradually improved, resulting in increasingly complex tissues in a dish [2,37]. Furthermore, researchers have been able to pinpoint particular organ areas, such as the brain and gastrointestinal tract [40,41]. Here, we review key studies that improved human iPSC organoid technology in disease modeling and drug discovery.

#### 2.2.1. Endoderm-Derived Organoids

PSCs were successfully differentiated into endoderm-derived organoids including, intestinal tissue containing functional enterocytes, goblet cells, paneth and neuroendocrine cells [42]. Using a tissue-engineering approach with embryonic and iPSCs Workman et al. generated human intestinal tissue organoids with a functional enteric nervous system [43]. By combining human-PSC-derived neural crest cells (NCCs) and developing human intestinal organoids (HIOs), their model recapitulated normal intestinal enteric nervous system development and thus it may be used to investigate the cellular and molecular basis for Hirschsprung’s disease in addition to screening for a new class of drugs to treat gastrointestinal disorders [43]. Zhang et al. also explored the potential of *CDX2* positive posterior gut endoderm progenitor cells that can form gut and liver organoids without the risk of teratoma generation when compared to iPSCs [44]. Onozato et al. reported the generation of intestinal organoids from cynomolgus monkey iPSCs. These cells were induced into the hindgut then populated on microfabricated culture vessel plates to generate spheroids that were differentiated into intestinal organoids in a medium encompassing small-molecule compounds.The generated organoids were also characterized by a polarized epithelium and were composed by numerous cells that made up small intestinal tissues. Interestingly they also expressed functional tight junctions drug transporter protein, which allows a broader biochemical investigation [45].

Human gastric organoids were established using temporal manipulation of key signaling pathways including FGF, WNT, BMP, retinoic acid, and EGF pathways and used for modeling infection with Helicobacter pylori [41]. Broda et al. also described two protocols to derive human gastric organoids that contain both the antrum and corpus compartments from human pluripotent stem cells (hPSCs) [46]. Biliary development and disease were modeled using cholangiocytes derived from iPSCs that generated 3D organoid systems. These cholangiocyte organoids could efflux bile acids in addition to their functional secretory role [47,48], allowing for the modeling of Alagille syndrome, a disorder characterized by impaired bile duct formation induced by Notch signaling disruption [48]. Also, in cholangiocyte organoids derived from iPSCs from patients with CF, CFTR corrector drug VX-809 rescued the disease phenotype of CF cholangiopathy [47,48]. Moreover, a vascularized and functional human liver was successfully established after transplantation of in vitro iPSC-derived organoids into mice [49]. Koike et al. used hPSCs-generated organoids to model the orchestration of organogenesis by boundary tissue interactions. In fact, it was shown that these boundary interactions between anterior and posterior gut organoids initiated a retinoic acid-dependent generation of hepato-biliary-pancreatic organ domains that are specified at the foregut–midgut boundary organoids without the influence of extrinsic factors [50].

It is worth noting here that as Min et al. highlighted in their review [51], it is important to acknowledge that in order to accurately emulate the gastrointestinal system with its relevant diseases, the in vivo microenvironment that is composed by the multitude of proximal cell populations (such as immune cells, fibroblasts, neural cells and the circulatory pathways) and the surrounding microbiota should be combined with gastrointestinal organoids.

Human iPSCs can be differentiated into functional thyroid tissue [52] which aids in cell-based regenerative therapy for hypothyroidism and in vivo production of circulating thyroid hormone [52].

Several studies reported the generation of lung organoids from hPSCs as useful tools to study lung development, maturation and disease [53,54]. Lung organoids were generated via differentiation of hPSCs using signaling pathways manipulation. hPSCs were grown to generate ventral-anterior foregut spheroids, which are then propagated to form human lung organoids (HLOs). These organoids bear a significant cytostructural similarity to the native organ as they hold both a mesenchymal and epithelial compartment. Histologically, a basal cell compartment along with immature ciliated cells that are enclosed by myofibroblasts and smooth muscle cells form an upper airway-like epithelium. An alveolar-like compartment is also present in these HLOs. RNA-sequencing was also employed to prove that the HLO share a common global transcriptional profile with the human fetal lung. The remarkable analogy and fidelity in modeling human tissue implies that HLOs can be employed as models to study human organogenesis and to assess novel therapies for lung and respiratory diseases [53]. In terms of methods, McCauley et al. also reported the differentiation of hPSCs to proximal airway epithelium. Cells are first subjected to definitive endoderm induction, then to a foregut anteriorization stage and finally they are restricted to *NKX2-1+* lung epithelial progenitors. The progenitor cells are ultimately cultured in a 3D environment with specific conditions to form the epithelial-only airway organoids [55]. Even more recently, Hawkins et al. derived airway basal cells from hPSCs. These cells were shown to be highly analogous to adult primary airway basal cells [56].

iPSCs derived from CF patients that are homozygous for the *F508del* mutation efficiently shaped proximal airway organoids associated with impaired forskolin-induced swelling. Forskolin-induced swelling was rescued by genetic correction of *F508del* in iPSCs, demonstrating the ability of gene editing to validate genotype-phenotype relationships [57]. Besides, surfactant processing in alveolar epithelial type 2 cells is restored after CRISPR-based gene correction of PSCs derived from patients with a homozygous surfactant mutation. As a result, AEC2s derived from PSCs provide a forum for disease modeling and possible distal lung functional regeneration [58]. Similarly, a homozygous deletion of *F508* in the CFTR gene was corrected in iPSCs from CF patients. Subsequently, recovery of normal CFTR expression was obtained as the corrected iPSCs differentiated to mature airway epithelial cells, suggesting an iPSC-based model system to develop new therapeutic approaches for CF patients [59].

As a final note, it is vital to mention that a considerable effort was dedicated to improving endoderm-derived organoid culture systems. For instance, Giobbe et al. suggested that extracellular matrix hydrogels derived from decellularized porcine small intestinal tissue can provide natural environment for the generation, stable growth, and in vivo transfer of endoderm-derived organoids [60]. The air liquid interface method that relies on collagen gels was recently employed to harbor the growth of 3D primary cells containing components of both mesenchymal and epithelial origin from mouse and human gastrointestinal tissues [61].

#### 2.2.2. Mesoderm-Derived Organoids

The discovery of hiPSCs aided in establishing mesodermal-derived organoids for disease modeling. Kidney organoids were successfully established from human iPSC to model human nephrogenesis and polycystic kidney disease (PKD) [62,63]. Takasato et al. described the generation of kidney organoids from hiPSCs. Interestingly, these organoids were constituted by all the expected kidney cell populations (glomerulus, proximal tubule, loop of Henle, distal tubule, collecting duct, endothelial system, and the interstitium). These organoids were highly analogous to the first trimester kidney based on transcription profiles. Protocol-wise, hiPSCs are differentiated into the posterior primitive streak, then the induction of the posterior and anterior intermediate mesoderm takes place. These will combine and self-organize to form the organoid [63,64,65].

Researchers revealed that iPSCs can be differentiated into ureteric bud progenitor-like cells that, when cultured in 3D, grow into primitive ureteric buds, which can be used to model PKD [66]. In addition, hiPSC-derived podocytes mimic Adriamycin-induced albuminuria and podocyte injury by reconstituting kidney glomerular-capillary-wall function on a chip [67]. Consequently, this will facilitate drug discovery and renders personalized medicine for diseased patients more effective.

Moreover, iPSC-derived cardiac organoids exhibiting fetal-like differentiation have been used to model cardiomyocyte regeneration following injury [68]. Also, iPSCs were used to engineer 3D cardiac muscle tissue to assess the effects of mechanical forces [69], metabolism [70], and the extracellular matrix [71] on cardiomyocyte maturation in addition to modeling Barth syndrome cardiomyopathy using 3D organ-on-a-chip technology [72]. Recently, Buono et al. generated triculture organoids by combining either healthy or hypertrophic cardiomyopathy-associated hiPSC-derived cardiomyocytes with microvascular endothelial cells and human cardiac fibroblasts. Their study reported distinct structural and beating activity variations in cardiomyopathic models compared to healthy organoids [73]. Also, hiPSC-derived cardiomyocytes were employed to model channelopathies affecting the heart, such as catecholaminergic polymorphic ventricular tachycardia [74], long QT syndrome [75], and QTS3/Brugada overlap [76]. Disorders affecting the structure, contractility, and survival, such as Duchenne muscular dystrophy [77], dilated cardiomyopathy [78], hypertrophic cardiomyopathy [79], Leopard Syndrome [80], Barth Syndrome [81], arrhythmogenic right ventricular cardiomyopathy [82] in addition to Torsade de Pointes [83], and other reentrant arrhythmias [84] have also been modeled. Also, using a multistep protocol, vascular organoids were generated from both human ESCs and iPSCs. In response to in vitro hyperglycemic condition and inflammatory cytokines, human blood vessels showed induced thickening of the vascular basement membrane while they imitated the microvascular changes in diabetic patients following their transplantation into diabetic mice [85].

Using embryoid body culture conditions, iPSCs were guided to differentiate into intermediate mesoderm and the Müllerian duct which gives rise to the female reproductive tract including the uterus, the oviduct, and the upper vaginal canal [86]. Endometrial organoids models could be used to investigate the embryo-endometrium interactions to improve assisted reproduction outcomes and prevent early pregnancy loss [87] in addition to modeling endometrial disorders among of which are infertility, pregnancy disorders, endometrial cancers, endometriosis, and Asherman syndrome [88].

#### 2.2.3. Ectoderm-Derived Organoids

Neural organoids have been used to model a wide range of neurologic, developmental, and psychiatric disorders, providing a wealth of information about the pathobiology of neurologic diseases. HiPSC-derived cerebral organoids were established and modeled microcephaly. It was found that premature neuronal differentiation has occurred in patient organoids which in turn explains the disease phenotype [89]. Neural organoids derived from iPSCs from patients with autism spectrum disorder showed normal early neuronal differentiation and overproduction of the transcriptional repressor *FOXG1* favoring the inhibitory GABAergic neuron fate over glutamatergic fate. This differentiation imbalance is suggested as a mechanism underlying autism pathogenesis [90]. Timothy syndrome which is a neurodevelopmental disorder that is caused by mutations in the *CaV1.2* calcium channel-interneurons was modeled using iPSC-derived ventral and dorsal forebrain organoids. Using this system, Timothy syndrome shows abnormal migratory saltations in addition to interneurons functionally integrating with glutamatergic neurons to form a microphysiological system [91]. Tuberous sclerosis complex is a multisystem genetic disorder caused by mutations in the TSC1 or TSC2 genes [92]. Organoids generated from patient-derived iPSCs proved that mosaic biallelic inactivation is needed for the formation of dysplastic cells and increased glia development during neural progenitor expansion [93].

Furthermore, iPSC-derived 3D brain organoids have been successfully used to model lissencephaly [94] and Miller-Dieker syndrome [95]. Also, PSCs derived from patients with multiple familial Alzheimer’s diseases generated brain organoids that recapitulated age-dependent-Alzheimer’s disease pathologies including amyloid aggregation, hyperphosphorylated tau protein, and endosome abnormalities. Treatment with b- and y-secretase inhibitors reduced aggregates formation and amyloid and tau pathology [96]. For Huntington’s disease, 3D cerebral organoids were employed to investigate the effect of the huntingtin mutation on neuronal development in terms of abnormal cell organization and acquisition of mature neuronal identities [97]. Looking upon Parkinson’s disease, iPSCs carrying *LRRK2 G2019S* mutation were isolated from patients to develop human neuroectodermal spheres which showed the synaptic dysfunction pathway to be the most altered pathway in Parkinson’s disease PDOs. Remarkably, gastrointestinal organoids were also derived from the same class of patients’ iPSCs, demonstrating variations in gene expression of patients’ intestinal cells relative to the controls [98].

In a similar approach to model neuromuscular diseases, when co-cultured with skeletal muscle cells from the same individual, hiPSCs-derived motor neurons generate neuromuscular junctions. These organoids could be used to screen therapeutics that enhance motor activity in patients with amyotrophic lateral sclerosis, and patients with traumatic spinal or muscle injuries [99,100,101].

Mammary differentiation was successfully directed to generate mammary-like organoids in 3D culture from human iPSCs. These organoids could be used to establish in vitro models to assess the effects of various factors on mammary cell transformation and breast cancer growth, as well as for personalized mammary tissue bioengineering [102,103].

Retinal organoids constituting laminated cytoarchitecture, apical-basal polarity, and the ciliary structure were developed from iPSCs for pathogenic modeling of human photoreceptors in vitro and retinal ciliopathies [104]. Also, retinal organoids serve to model X-linked juvenile retinoschisis [105], and late-onset retinitis pigmentosa [106] and recapitulate the pathogenesis of retinitis pigmentosa, allowing for CRISPR-Cas9-mediated correction of RPGR mutation to reverse ciliopathy and photoreceptor loss [107].

### 2.3. iPSCs and Disease Modeling

#### 2.3.1. iPSCs and Cancer

Studying inherited human cancer syndromes has become more feasible with iPSCs methodologies. In an in vitro model of Li-Fraumeni syndrome- associated osteosarcoma, iPSC-derived from patients generated osteoblasts that recapitulated osteosarcoma features and exhibited decreased H19 gene expression relative to wild-type controls [108]. Ectopic expression of H19 expression in Li-Fraumeni syndrome osteoblasts aided osteoblast differentiation and suppressed tumorigenic potential, elucidating the novel role for the H19-Decorin pathway in suppressing osteosarcoma [108].

Familial adenomatous polyposis is an inherited disorder characterized by germline mutations in the *WNT* pathway regulator APC causing an increased chance of developing precancerous polyp in the large intestine [109]. To study colorectal cancer, colon organoids (COs) were established using patient-derived FAP-iPSCs [110,111]. The derived COs display colon features as they contained stem cells, transit-amplifying cells, goblet, and endocrine cells (ECs) [111]. Also, they exhibited enhanced WNT activity and increased epithelial cell proliferation [110,111] and were used for XAV939 and rapamycin drug testing [111].

In a study performed by Huang et al., PSCs were successfully differentiated to pancreatic exocrine lineage mainly through TGF-β and notch inhibition to generate human pancreatic adenocarcinoma (PDAC) organoids [112]. iPSC-derived PDAC organoids were used to gain clinically important insights into PDAC as they maintained tumor-specific traits, inter-patient variation in tumor histoarchitecture, and showed differential responses to EZH2 inhibition as a therapeutic approach against PDAC [112].

Tumors can be induced in iPSC-derived human cerebral organoids mainly through *RAS* activation and *TP53* deletion simultaneously [113]. In their study, Ogawa et al. not only reported the invasive phenotype of tumors generated in human cerebral organoids but also them being transplantable from organoids to mice and from organoid to organoid. Consequently, they demonstrate that organoids are good platforms to study the role of regulatory genes in addition to featuring tumor features and supporting the growth of primary human brain tumor explants [113].

Also, iPSCs isolated from patients with germline mutations that cause familial cancer predisposition syndromes modeled diseases such as Fanconi anemia [114], hereditary platelet deficiency with acute myeloid leukemia predisposition [115], and breast cancer predisposition [116]. Human iPSCs have also been used to model myeloid malignancies including juvenile myelomonocytic leukemia [117] and chronic myelomonocytic leukemia (CMML). Using CMML-iPSCs, Taoka et al. generated a humanized CMML mouse model and identified a MEK inhibitor, a Ras inhibitor, and liposomal clodronate as potential drugs for treating CMML [118].

#### 2.3.2. iPSCs and Infectious Diseases

Human intestinal organoids have been used to study viral infections including Middle East Respiratory Syndrome-related Coronavirus [119] and norovirus [120]. Also, organoids derived from human PSCs have been used to simulate epithelial tissue infection such as the infection of gastrointestinal viruses [121]. Using stem-cell induced human intestinal organoids (iHIOs), Finkbeiner et al. reported that rotavirus not only infected the epithelial cells but also the mesenchymal cell population of the iHIOs [121]. Similarly, hiPSC-derived HIOs have been employed in modeling host-pathogen interaction in *S. Typhimurium* infection [122].

Also, iPSC-derived lung bud organoids (LBOs) that contain mesoderm and pulmonary endoderm in addition to branching airway and early alveolar structures were employed to model infection with the respiratory syncytial virus. The infected epithelial cells display swelling and shedding, as seen in human lungs [54]. These findings show the ability of LBOs to recapitulate lung development suggesting the potential use of this model to recapitulate fibrotic lung disease.

On the other hand, human iPSC-derived cells are used as targets for pathogenic viral infection. Natural killer (NK) cells, a key component of the innate immune system, were successfully derived from hiPSCs. iPSC-derived NK cells exhibited potent anti-HIV-1 activity against target cells through direct lysis, antibody-dependent cellular cytotoxicity, and the production of soluble mediators [123]. Also, the introduction of the *CCR5del32* mutation into hiPSCs using CRISPR–Cas9 resulted in differentiated monocytes resistant to HIV infection [124]. Consequently, iPSCs provide an excellent model for cellular immunotherapeutic studies for the treatment of HIV/AIDS and human lymphocyte development. In a model of herpes simplex virus 1 (HSV-1) encephalitis, iPSC-patient-derived neurons were more vulnerable to HSV-1 infection than the controls [125]. Moreover, human iPSC-derived macrophages were used also to model Dengue virus and Zika virus (ZIKV) infection [126]. HiPSC-derived hepatocytes provide specific models for studying infectious diseases such as hepatitis B (HBV) and hepatitis C (HCV) [127,128,129,130]. HCV and HBV entry receptors have been identified in hiPSC-derived hepatocytes, indicating that they may support productive HCV or HBV particle infection [127,131,132].

## 3. Organoids from Embryonic Stem Cells (ESCs)

### 3.1. ESCs versus ASCs in 3D Culture and Disease Modeling

ESCs are briefly present in the early human or mouse embryo, a few days after fertilization. These ESCs have two major and crucial characteristics: Indefinite renewal and pluripotency. They can be grown indefinitely and have the potential to give rise to every tissue in our body. ASCs are undifferentiated cells among differentiated cells in a tissue or organ. They reside in specified microenvironments, called the stem cell niche. While ESCs can become all cell types, ASCs have a limited differentiation potential. The primary roles of ASCs are to maintain tissue homeostasis and to some extent, to replace cells that die due to injury or disease [133,134,135,136].

Both types of stem cells can be used to establish organoids for the central nervous system, kidney, and gastrointestinal organs. Studies have reported that adult epithelial stem cells carrying the generic Lgr5 marker cultured under tissue-repair conditions have been able to generate epithelial organoids from normal and affected organs like the pancreas, the liver, the gut, and the lung [135,137]. Moreover, we can apply to organoids several established approaches such as, single-cell RNA sequencing, mass spectrometry and cryo-electron microscopy [2]. Nevertheless, there are differences between ESC- and ASC-derived organoids in generating model systems. ESC-derived organoids establish structures via processes taking place only throughout embryonic development, in order to restate the in vivo development [138]. Generally, a multistage protocol is needed to extend and differentiate ESCs, and for each stage, a particular cocktail of growth factors is required. Each type of tissue’s differentiation needs a specific timespan, yet usually, it takes approximately two to three months to be achieved [139]. ESC-derived organoids are complex and might include mesenchymal, epithelial, and, in certain instances, endothelial constituents. Also, they are great models for investigating the development, genetic disorders, and infectious diseases, especially for organs with no or slight regenerative ability like the brain [139]. ESC-derived organoids were mainly established for the brain and then for other organs, such as the stomach, liver, intestine, lung, and kidney [37]. However, all ASC-derived organoids just show the epithelial parts of organs, subsequently, stroma, nerves and vasculature are absent, indicating that ASC-derived organoids are less complex than ESC-derived organoids. On the other hand, instead of modeling development, ASC-derived epithelial organoids are involved in adult tissue repair. Therefore, ASC-derived organoids may be developed only from tissue parts with regenerative ability [137]. In theory, ASC-derived organoids can be established from normal as well as from affected epithelial tissues within around one week after seeding of the cells [8,140].

### 3.2. Human and Mouse ESCs in 3D Culture and Disease Modeling

#### 3.2.1. Origin, Properties and Derivation

Here we highlight the advancement in ESCs derivation and their therapeutic potential in regenerative therapy and disease modeling. ESCs are pluripotent cells characterized by their self-renewal ability and potency to generate all the embryo’s somatic cell types. Their indefinite self-renewal ability and plasticity facilitate the limitless generation of different cell types in vitro, making them ideal candidates for regenerative therapy and disease modeling [16,141,142]. In 1981, murine ESCs (mESCs) were isolated for the first time from the inner cell mass (ICM) of a mouse blastocyst. Like human ESCs (hESCs), mESCs exhibit self-renewing capacity, in addition, to their differentiating potential into any somatic cell lineage [143,144]. However, in 1998, the first hESCs were isolated from in vitro fertilization blastocysts [145] which marked the beginning of a modern era of regenerative medicine.

hESCs are collected from early-stage human blastocysts [17], a structure produced after multiple mitotic cell divisions following the formation of a diploid zygote [141] or from tissues at later stages (3 months or less gestational age) [17]. Production of hESCs lines from the inner cell mass at the blastocyst level, has raised ethical and political issues as it requires the destruction of the embryo [141]. To deal with this issue, a lot of research has gone into isolating cells from earlier stages of embryonic development without destroying the embryo either from single blastomeres [146,147] or morula stage [148]. Unfortunately, blastomere differentiation to ICM was inefficient due to the blastomere-derived aggregates that mostly gave rise to trophectoderm-like vesicles. However, the efficiency of blastomere-hESC derivation has been improved by adding laminin to the culturing media [149]. Laminin, a component of the basement membrane [150], recapitulates the ICM niche preventing the polarization of the blastomeres into ICM and trophectoderm [149]. The first method for successfully establishing hESCs lines was to co-culture isolated stem cells with mouse embryonic fibroblasts (MEFs), which also have differentiation capabilities to other cell types [151,152,153,154]). As reported by Thompson et al., the hESCs were grown on MEFs feeders in a medium containing fetal bovine serum. The generated hESCs were karyotypically normal and generated all germ cell teratomas in vivo [145]. However, the use of animal-derived products renders hESCs lines unsuitable for human cell transplantation due to possible transfer of pathogens that could be transmitted to patients including retroviruses in mouse embryonic fibroblasts [155]. Furthermore, mouse feeder layers are a source of N-glycolylneuraminic acid (Neu5Gc), a nonhuman sialic acid that could be uptaken by the hESCs grown on these layers [156]. Consequently, hESCs-derived tissues expressing Neu5Gc may trigger an immune response due to the natural antibodies to Neu5Gc present in most humans leading to a compromised transplantation success [156]. Therefore, to reduce the xenogenic contamination from animal feeder cells and sera, xeno-free conditions were employed to derive hESCs lines using human feeder cells and serum replacements or serum-free conditions [157,158,159] in addition to human serum [160,161]. Therefore, hESCs lines derived under xeno-free culture media conditions can meet the criteria for both clinical applications and transplantation purposes. Alternatively, hESCs can be maintained in feeder-free environments using extracellular matrices such as matrigel and 3D culture models [162,163].

#### 3.2.2. hESCs-Derived Organoids

hESCs have tremendous potential for stem-cell-based therapy of human diseases. Moving from traditional 2D to 3D cultures facilitates the generation of physiological in vitro models of human development and disease. ESCs-derived organoids have emerged as an effective technique to study human diseases and possible treatments. Leibel et al. describe a protocol allowing the differentiation of hESCs into 3D structures, harboring epithelial and mesenchymal cells, as well as ciliated cells, and producing surfactant. These 3D arrangements mimic the development, architecture, and function of the lung. These lung organoids could be used to evaluate the effect of different toxins and to test drugs. This model can then be used to investigate lung disease due to respiratory viruses such as SARS-CoV-2 [164].

Historically, insulin producing islet cells are one of the earliest functional tissue engineered organoid concepts. Given their ability to differentiate into hormone-producing pancreatic β cells [165,166,167], hESCs-derived ECs form pancreatic islet-like organoids that mimic pancreatic islets regarding β cell maturation, glucose responsiveness, and blood glucose level control in diabetic mice [168]. Interestingly, hESCs-derived EC clusters showed faster regulation of glucose level in vivo compared to hESCs-derived ECs in addition to expressing endocrine hormones including insulin, c-peptide, somatostatin, and pancreatic polypeptide [168]. Wang et al. described the formation of islet organoids (IOs) from H9 human ESCs in biomimetic 3D scaffolds. The IOs were formed by pancreatic alpha, beta, delta, and polypeptide cells. In fact, these IOs expressed mature beta cell markers. When it comes to their endocrine function, IOs highly expressed C-peptide which indicates that a *de novo* endogenous insulin synthesis was taking place. IOs could also be considered as mature beta cells as they expressed insulin-secretory granules. Additionally, the fact that IOs secrete insulin when exposed to a high glucose concentration further support this notion [169]. Other research groups displayed similar efforts when it comes to generating “pancreatoids” [170]. Collectively, these findings suggest the use of EC clusters derived from human PSCs as a therapeutic approach in diabetes mellitus.

Moreover, using a two-step protocol, hESCs—derived expandable hepatic organoids (hEHOs) were allowed to expand in vitro followed by the induction of their hepatic maturation in serum and feeder culturing conditions [171]. In the same study, an ex-vivo liver system was generated upon incorporating human fetal liver mesenchymal cells into the hEHOs. This system provides a reliable model to study the pathophysiological changes, pathogenesis and therapeutics in alcoholic liver disease and allows to model fatty liver disease such as steatohepatitis through introducing Kupffer cells to the derived system [171,172].

Differentiation of hESCs into human prostate organoids was accomplished to investigate the effect of Bisphenol A (BPA) on the human fetal prostate [173]. Activin A was used to induce hESCs- differentiation towards prostate epithelium and stroma. Besides, a cocktail of WNT10B and FGF10 and appropriate media was included to promote the formation and maturation of prostatic organoids which recapitulated the key prostate developmental events as they occur in utero. The results show BPA exposure modifies prostate organoid branching and increases stem-cell-like numbers in mature organoids, indicating that low-dose BPA in utero impairs the growth of the human fetal prostate [173].

The loss of midbrain dopaminergic (mDA) neurons is known to be a key pathological characteristic of Parkinson’s disease [174]. Therefore, wide research has concentrated on generating mDA neurons from hESCs in recent years. Lately, a method for differentiating hESCs into human midbrain-like organoids (hMLOs) was reported. These hMLOs recapitulate characteristics of the midbrain and might be valuable as a model for investigating midbrain function and dysfunction [175].

On the other hand, human cortical organoids (hCOs), generated from hESCs present an efficient tool to study human brain development and diseases in 3D structures [176]. Yet, present-day hCOs lack microvasculature, leading to limited oxygen and nutrient transport to the inner-most parts of hCOs. A recent protocol used hESCs ectopically expressing human ETS variant 2 (ETV2). ETV2-expressing cells in hCOs helped to create a complex vascular-like network in hCOs. The presence of vasculature-like structures yielded increased functional maturation of organoids. These vascularized hCOs (vhCOs) have many blood-brain barriers features, such as an increased expression of tight junctions, nutrient carriers and trans-endothelial electrical resistance. By mirroring the vasculature in the early prenatal brain, these vhCOs show a strong model to study brain disease in vitro [177].

Recently, a 3D artificial thymic organoid model was reported to induce successful differentiation of hESCs to mature, functional, conventional T cells in vitro, with a varied T cell receptor repertoire. This method is relevant to the evaluation of human T cell development, and the establishment of likely adoptive T cell immunotherapies for cancer [178].

#### 3.2.3. mESC-Derived Organoids

Tan et al. published a protocol in which mESCs are differentiated into ureteric bud progenitor cells. When these cells are co-cultured with primary metanephric mesenchyme, nephrogenesis is initiated [179]. Kidney organoids that functionally recap kidney-specific physiology and constitute proximal tubules, podocytes and endothelium were derived from mESCs [62]. Upon chemical injury, tubules in the derived model expressed kidney injury molecule-1 (KIM) which provides a quantifiable human criterion for predicting proximal tubule nephrotoxicity. Also, these organoids allowed examining both the defects in junctions organization in response to knockout podocalyxin and cystogenesis in PKD [62]. A recent study reported an assemblage of kidney organoids that mimics physiological interaction and embryonic branching morphogenesis. A protocol was established to differentially induce each lineage from mouse ES cells. Assembled organoids developed the peripheral progenitor niche as well as the differentiated nephrons which are linked by a ramified ureteric epithelium, constituting the fundamental architectures of the embryonic kidney [180].

Functional thyroid follicles were successfully derived from mESCs and generated functional thyroid organoids that recapitulate the morphological and functional properties of *bona fide* thyroid follicles. Following their transplantation into athyreoid mice, ESC-derived thyroid follicles developed functional thyroid tissue which rescued thyroid hormone deficits in vivo [181].

Knowing that the human stomach can be divided to two main compartments, the antrum, and the corpus, it is primordial to ensure that gastric organoids should be able to reflect a relatively accurate representation of these compartments. Noguchi et al. described the generation of such gastric organoids from mESCs. The authors employed an embryoid body-based differentiation technique to generate gastric primordial epithelium that is enclosed with a mesenchymal compartment. The resulting epithelium was further cultured in a 3D environment [182].

mESCs-derived embryoid bodies were employed to develop heart organoids containing cardiac muscle, conducting tissues, smooth muscle and endothelial cells that showed myocardial contraction and action potentials [183]. Also, cardiomyocytes derived from hESCs cells proved to be useful in cardiac regeneration and in enhancing cardiac function in myocardial infarctions [184,185,186].

Recently, an in vitro model was developed for the derivation of inner ear sensory epithelia from mESCs, using a step-by-step signaling molecules treatment. The resulting tissue includes functional hair cells as well as a neuronal population innervating the derived epithelia. This in vitro inner ear organoid culture system might help as a precious tool in disease modeling, developmental and physiological research, drug testing, and other potential cell-based therapies [187]. Recently, a study reported for the first time to use the embryoid bodies to form 3D spheroids of otic organoids [188]. The hanging-drop method, does not require any synthetic reagents or force apart from gravity [189].

Furthermore, a therapeutic approach based on light energy “Photobiomodulation” (PBM) (also known as a low-level laser therapy, which is known to stimulate the migration, the proliferation and the differentiation of stem cells [188]), was used during “otic organoid” generation from these mESCs. Inner-ear hair cell-like cells differentiation was enhanced by PBM. This study is helpful on the level of disease modeling and improving present differentiation protocols to generate inner-ear hair cells [188].

The orthotopically functional salivary gland was successfully generated by using the transplantation of an induced salivary gland primordium (iSG) from mouse ESCs. The iSGs secreted saliva after orthotopic transplantation in mice, by the restoration of neural network in vivo [190].

Recently, Lee et al. generated skin organoids, harboring epidermal and dermal layers, in vitro from mouse ESCs, under serum-free conditions. Furthermore, these skin organoids can spontaneously give rise to *de novo* hair follicles in a process that reproduces normal embryonic hair folliculogenesis. Hence, this in vitro 3D model of skin development will help study the mechanisms of hair follicle induction, assessing hair growth, testing drugs, and finally modeling skin diseases [191].

### 3.3. ESCs in Disease Modeling

#### 3.3.1. ESCs and Genetic Diseases

Human ESC lines harboring mutations are valuable models to imitate genetic diseases and the development of new therapeutic strategies. For instance, Lesch-Nyhan syndrome is characterized by the accumulation of uric acid is caused by a mutation in the HPRT1 enzyme [192]. Using homologous recombination, HPRT1 mutation is introduced in human ESCs which modeled Lesch-Nyhan syndrome and recapitulated the high rate of uric acid accumulation [193]. Also, the development of pancreatic beta cells from hESCs carrying mutations that cause monogenic forms of diabetes such as maturity-onset diabetes of the young and insulin resistance syndromes [194] will facilitate their modeling and further discovery of a cure.

#### 3.3.2. ESCs and Regenerative Medicine

Existing organoid technologies will also assist future retina research and regenerative medicine. Retinal organoids were established from mESCs and hESCs to generate a coordinated, complex, stratified 3D retinal tissue, which reproduces the dynamic, and temporally regulated retinogenesis [195]. In their study, Völkner et al. indicate that the inhibition of Notch signaling allowed to enrich retinal organoids with cone or rod photoreceptors. Consequently, these findings will promote ESC-based models in various applications including cell replacement therapy.

Recent research has shed light on the ability of ESC-derived neurons to form functional connections in the brain and spinal cord in a variety of lesion and disease models. Using a combination of retinoic acid and Sonic hedgehog signaling, Wichterle et al. generated mESCs- derived spinal cord motor neurons that innervate multiple muscle targets following transplantation to the developing chick spinal cord [196]. Also, Peljto et al. revealed the generation of brachial and thoracic, limb innervating motor neurons through intraspinal transplantation [197]. Similarly, hESCs were employed to develop engraftable spinal cord motor neurons with both rostral and caudal motor neuron features [198]. On the other hand, several studies brought out the generation of transplantable cortical progenitors from mouse and human ESCs which gain forebrain individuality upon neural induction [199]. In several spinal cord injury patients, a loss of GABAergic tone in the injured spinal cord can lead to neuropathic pain and bladder dysfunction [200]. However, the adverse effects of spinal cord injury are ameliorated following transplanting hESCs—derived medial ganglionic eminence (MGE)-like cells into injured mouse spinal cords. MGE differentiation into GABAergic neuron subtypes that functionally integrate into the host spinal cord results in reduced pain and alleviated neurogenic bladder dysfunction [201]. Altogether these studies highlight the capacity for ESC to restore central nervous system (CNS) functionality and circuitry. An alternative approach has evolved by converting ESCs into neural progenitor cells or cell lines. HESCs can differentiate into neural cell types including neurons, oligodendrocytes, and astrocytes, elucidating their role as a source of transplantable neural precursors for nervous system repair and treatment for neurological disorders [202,203]. Also, their potency to generate cardiomyocytes [204], cells with characteristics of insulin-producing β-cells [205], osteoblasts [206], and hemopoietic progenitors [207] indicates the promising use of hESCs in clinical applications and regenerative therapy.

#### 3.3.3. ESCs and Cancer

Preclinical cancer models are essential to investigate cancer biology, in particular tumor heterogeneity. One of the interests of the organoid culture model is the use of similar culture techniques for normal and neoplastic tissues. Under defined conditions, tumor organoids (Tumoroids) can be successfully established and propagated from tumor tissue such as patient biopsies which mimic the original cancer tissue [8,208].

As proof of the concept, a malignant model of Retinoblastoma (Rb) in retinal organoids generated from gene-edited hESCs with biallelic mutagenesis of the *RB1* gene. These organoids show features highly compatible with Rb tumorigenesis, transcriptome, and genome-wide methylation. Mainly, the authors found aberrant deregulation of the PI3K-Akt pathway and a significant up-regulation of its activator spleen tyrosine kinase (SYK). Furthermore, SYK inhibitors induced exceptional cell apoptosis in tumor organoids [209].

On the other hand, a study developed a 3D in vitro model to replicate brain metastasis using hESCs-derived cerebral organoids (metastatic brain cancer cerebral organoid [MBCCO]). The MBCCO model efficiently reproduced metastatic cancer processes, such as cell adhesion, proliferation, and migration, as well as cell-cell interactions. The authors show that lung-specific X protein is highly involved in cell proliferation and migration. Moreover, they noticed astrocyte accumulation around and their interaction with malignant cells through connexin 43. Furthermore, they assessed the effects of gefitinib, a common anticancer agent. Thus, the MBCCO model is a robust tool for modeling human metastatic brain cancer in vitro [210].

#### 3.3.4. ESCs and Infectious Diseases

Organoids’ properties also make them a powerful model to study the interaction between a host and infectious organisms such as *Helicobacter Pylori* or *Salmonella Enteritica*, gut-microbiota interactions, and inflammatory bowel disease, as well as the resulting inflammatory conditions. Furthermore, patient-specific responses to microbes can be mainly exploited [211]. The interaction between *Clostridium difficile* (*C. difficile)* and complex human epithelium was studied by using the HIOs. Viable *C. difficile* was introduced by microinjection techniques into the lumen of HIOs. Colonization of HIOs with *C. difficile* strain *VPI 10463* leads to the disruption of the organoid epithelium. These effects seem to be caused by the primary virulence factors of *C. difficile*, the toxins TcdA and TcdB. This study shows that HIOs can be used for the comprehensive molecular and cellular analysis of the pathogenic interactions between *C. difficile* and human intestinal epithelium [212].

The link between ZIKV infection and microcephaly was investigated by using hESCs-derived cerebral organoids to recapitulate the early stage of fetal brain development. The authors show that a prototype strain of ZIKV, MR766, successfully infects organoids and leads to a decrease in overall organoid size that relates to the kinetics of viral copy number. Furthermore, they observed the upregulation of the innate immune receptor Toll-like-Receptor 3 (TLR3) after ZIKV infection of human organoids and mouse neurospheres. On the other hand, TLR3 inhibition decreased the phenotypic effects of ZIKV infection [213].

## 4. Organoids from Adult Stem Cells (ASCs)

### 4.1. ASCs in 3D Culture and Disease Modeling

#### 4.1.1. Origin, Properties and Derivation

ASCs, also known as somatic stem cells or tissue-specific stem cells, are a rare population of multipotent cells with regenerative and self-renewal capacities. They play a role in tissue homeostasis and replacement of damaged and dead cells. Stem cells resided in stem cell niche, a specific microenvironment that provides the essential factors to regulate cell fate [134]. ASCs have been found in several tissues, including brain [214,215], bone marrow [216,217], liver [218], adipose tissue [219], intestine [220,221,222], and skin [223]. With aging, the number, regenerative ability, and growth of these cells decreases [224,225]. As previously mentioned, while ESCs are pluripotent and can generate all cell types, ASCs are multipotent or unipotent, meaning they can only yield distinct cell types [226]. ASC-derived organoids are generated directly from postnatal or adult tissue through a process that involves stepwise differentiation protocols and requires activation of various signaling pathways mediated by intrinsic and extrinsic cellular factors. Beside normal tissues, ASC PDOs are a useful tool for disease modeling and precision medicine [13].

#### 4.1.2. ASC-Derived Organoid Models of Normal and Diseased Human Tissues

Human ASC-based organoids have been used to model several diseases, including cancer, infectious diseases, and inheritable genetic disorders. There have been many studies over the past decades, which have investigated signaling pathways through modeling human disease biology. This has provided an extensive understanding of adult tissue maintenance and repair [227].

Due to the genomic instability and limited amount of tissue identities within HeLa cells and other immortalized human cell lines [228], the focus of recent studies switched *to* in vitro models derived from stem cells. This has permitted a wider array of tissue identities to be identified, enhanced genomic integrity, long-term expansion, and improved modeling of healthy biology. Traditionally, studies using human disease tissues had constraints related to availability of the native disease tissue, restricted accessibility to the tissue, and ethical concerns. The fact that organoids can be established from a small amount of tissue and can be rapidly and effortlessly cultured makes them adequate models for studying multiple diseases [229].

In 2009, the stem cells field has witnessed a major methodical advance by the successful development of the intestinal organoid culture system referred to as the R-spondin method [230]. The tumor microenvironment contains tumor cells and stromal cells supporting cancer development. These ASC-derived organoids are embedded in a matrix simple, called Matrigel supplemented with different growth factors establishing key endogenous niche signals: WNT, a Frizzled/LRP (lipoprotein receptor-related protein) ligand; Noggin (an inhibitor of bone morphogenetic protein) for stem cell expansion; R-spondin (a WNT agonist) for maintaining stem cell populations; and EGF for promoting cell proliferation. This system was used to create 3D structures with distinct crypt-like and villus-like domains [3]. Remarkably, these organoids dependably recapitulated the in vivo tissue architecture. The system was subsequently adapted for not only generating human intestinal organoids, but also organoids derived from other organs, particularly from colon and stomach having Lgr5+ stem cells [230,231,232,233]. This first report of establishing 3D organoid culture derived from a single ASC was followed by many subsequent organoid works in mesendoderm (e.g., stomach, liver, pancreas, lung, and kidney) and neuroectoderm (brain and retina) systems [230,231,232,233,234].

Although the earliest 3D epithelial organoid models were first described over 40 years ago, their limited in vitro viability prevented them from being used in translational medicine. These drawbacks are now largely excluded along with advances and extensive research on multipotent stem and progenitor cell isolation. In 2009, Sato et al. [230] were able to produce indefinite expansion of self-renewing intestinal organoids. The proposed method conducted intestinal crypts and FAC sorted Lgr5+ intestinal epithelium cells embedded in a 3D matrix called Matrigel [230]. After optimizing this method, we are now able to produce organoids from human colon and colorectal adenoma tissue noting that the Clevers group are the ones who derived epithelial organoid models from human colorectal cancers [230,235,236]. The characteristics of these 3D epithelial organoids are multipotent cellular differentiation, biologically relevant cellular signaling, and a complex intercellular communication and organization network.

In a culture medium supplemented with EGF, noggin and the Wnt agonist R-spondin (RSPO), intestinal organoids were able to be established. The protocol was developed by embedding the intestinal stem cells in a 3D Matrigel. The protocol followed is now required during in vitro tissue development and in adult homeostasis and repair. By merging the knowledge on stem cell populations and utilizing information from the intestinal stem-cell niche requirements, intestinal organoids from either postnatal or adult intestinal epithelium [237] or from a single adult intestinal stem cell [230] were established. Similarly, mouse- and human-derived colonic stem cells have also been expanded into organoid accompanied with slight modifications in the medium composition and culture conditions [231]. Sato et al. [231] have succeeded in the long-term expansion of adult intestinal epithelium in culture while retaining its characteristic budding structure and displaying all the hallmarks and architecture of all individual derivatives [231,237].

Gastric organoids are established either by expansion of adult gastric stem cells from both the corpus and the antropyloric epithelia, or from differentiation of PSCs. After the identification of the marker for pyloric stem cells *Lgr5* [232], development of the first long-term culture system of mouse gastric stem cells took place. The expansion of human gastric organoids from corpus and pylorus stem cells was enabled by the blockade of transforming growth factor-beta (TGFβ) signaling [140]. These gastric organoids were grown in Matrigel, essential for organoid development, and in medium culture containing WNT3A and FGF10 [232,233]. Noggin, the BMP antagonist, is a critical factor that prevents intestinalization and promotes a foregut fate. The differentiation has shown acid-producing parietal cells are to be more difficult to develop [238]. Spurrier et al. were the first to successfully generate a human tissue engineered esophagi that contain both an epithelial and mesenchymal component. Esophageal organoid units (EOUs) were extracted from human or murine esophagi and transferred on a polyglycolic acid/poly-l-lactic acid collagen-coated scaffold to adult immune-deficient or allogeneic mice. The resultant engineered esophagi were composed of both the epithelium and inferior muscular layer. Even when the murine EOUs were cultured in vitro, they expanded as a sphere of proliferative basal cells in proximity to a neuromuscular system that exhibited spontaneous peristaltic movement [239]. These findings suggest that the idea of successfully growing human esophagi on a biodegradable scaffold can be further exploited for therapeutic purposes.

Human brain organoids were successfully established and are now used to model human brain development and disease. Microcephaly was the first disease studied in these models [39]. In addition, these models were used to determine the relationship between the ZIKV and microcephaly and the effect of this virus on brain development [213,240]. Brain organoid model systems also allowed for a comparison in the virulence of the African and Brazilian strains [240] and were used to identify the viral protein that inhibits neural stem cell proliferation [241]. Importantly, compounds that inhibit ZIKV infection and alleviate the hypomorphic effect of the virus have been also identified using the human brain organoid models [242].

Bone spheroids known as “osteospheres” are new in vitro models to study the molecular mechanisms of bone remodeling. A promising approach to form osteospheres from human adult bone precursor cells was reported by Kale et al. [243] The formation of osteoblast requires 3D aggregation of the cells, removal of serum with the presence of TGFβ1. This current approach lacks the varied cell types and tissue architecture. Further studies are needed to see whether improvements in 3D culture methods could further develop these spheroids to bona-fide bone organoids.

As previously mentioned, our group isolated PDOs from prostate tissue [8]. Human fetal adrenal cells were also used to generate adrenal organoids that had shared morphofunctional characteristics with the fetal native gland [244]. Human trophoblast organoids generation was also reported in the literature. Such organoids can serve as models to investigate placental development and to study the delicate fetal-maternal interaction [245]. It is interesting to note that it is possible to incorporate mesodermal progenitor cells into other organoids, thus forming complex organoids. This can further enhance the faithful representation of native organs as such organoids would have vascular networks [246].

Although replicating the distinctive extracellular matrix that thymic epithelial cells require has been a major constraint to culturing thymic organoids, Tajima et al. reported the generation of thymic organoids from decellularized thymus scaffolds that were repopulated with selected thymic epithelial cells. These scaffolds maintained the 3D microenvironment that can sustain the growth of the epithelial cells. The organoids were successfully populated by lymphocyte progenitor cells when transplanted into athymic nude mice [247]. Since thymic transplants in athymic patients (such as pediatric patients with complete DiGeorge anomaly) are limited by post-transplantation immune dysregulation and an improper T-cell “education”, Valente et al. postulated that organoids may help in overcoming these obstacles. After transplanting athymic mice with a microporous annealed particle scaffold structure seeded with thymic stromal cells, it was shown that T-cell repopulation was achieved as early as 2 weeks [248]. Based on these findings, it is possible to postulate that modulating T-cell adaptive immunity within the realm of regenerative medicine is not a far-fetched concept.

Lung organoids describes both upper- and lower-airway organoids. Establishing organoids from adult lung tissue has been a difficult process. Hogan and colleagues [249] were able to pioneer upper-airway organoids studies by which the single basal cells would become bronchiolar lung organoid cultures. The protocol that was followed by them has proven limited expansion potential yet were able to differentiate to both basal and luminal cells [249]. The generation of distal (lower)-airway lung organoids has been furthermore difficult. Alveoli consist of surfactant-secreting type II (AT2) and gas-exchanging type I (AT1) cells. Likewise, Barkauskas et al. were able to establish self-renewing human alveoli by co-culturing human AT2 cells with lung fibroblasts [250].

Human dental stem cells (hDPSCs) have mesenchymal (stromal) stem cell-like properties and express multiple conventional MSC markers. Their gene expression profile is similar to that of bone marrow MSCs. hDPSCs are used in dental tissue engineering and can be developed into iPSCs [251]. Gronthos et al. [252] firstly reported the isolation of dental pulp stem cells (DPSCs) from human impacted third molars. Jeong et al. [253] were the first to fabricate a novel dentin-pulp-like organoid from hDPSCs, which are mesenchymal stem cells, under different culture conditions. Briefly, hDPSCs were mixed with Matrigel and transferred into the maintenance medium and odontogenic differentiation medium containing supplements and essential growth factors. Gene expression and histology analysis demonstrated that the established organoids showed characteristics of both stem cells and differentiated odontoblast-like cells. This was also accompanied by mineralization and odontoblastic differentiation, in addition to proper response to a biological stimulator. The described dentin-pulp-like organoid fabrication procedure is a promising tool for future therapeutic strategy for tooth regeneration [253]. A 3D biofabrication system, the magnetic 3D bioprinting was also used to generate organoids from hDPSCs. The protocol involves tagging cells with magnetic nanoparticles before culturing them in Matrigel. hDPSCs were enriched for specific salivary gland epithelial progenitors and were used to generate SG-like organoids [254].

### 4.2. ASCs in Disease Modeling

#### 4.2.1. ASC-Derived Organoids and Cancer

Organoids can be directly derived from diseased tissue, allowing an expansion of diseased epithelium of the common tissue origin, dealing with a wide range of diseases, and overcoming limitations attributed to scarcity of disease tissues. PDOs are used in cancer research as a platform to predict the efficacy of a wide range of anticancer drugs and therapeutics. Multiple organoids have been established from gastric [255,256,257], colorectal [258,259,260,261,262], brain [263,264], pancreatic [265,266,267], liver [268], bladder [269], prostate [270], breast [271], esophagus [272], lung [273,274], and endometrial [275] cancers. The successful derivation of organoids from these tissues is highly dependent on sample quality and volume, however the rate of organoid derivation from tissues is far exceeds the probability of forming stable cell lines from the same tissues. These organoids often resemble the histological characteristics of the tissue they are derived from. For instance, patient-derived papillary thyroid cancer organoids were successfully isolated in a 3D environment by Chen and colleagues. Authors demonstrated that these organoids have faithful genomic and histopathological profiles to the native neoplasms. The drug sensitivity assays that were conducted on these PDOs also resulted in patient-specific drug responses that were heavily correlated with the corresponding tumor mutational profile [276]. Nevertheless, in their recent systematic review about thyroid organoid models, Samimi et al. highlighted the lack of studies that employ organoids as a tool to understand thyroid tumorigenesis. Despite this limitation, the authors admitted that such organoid models may provide an exceptional preclinical in vitro simulation [277].

Recent large cohort studies demonstrated that organoids derived from tumor tissues served as tools to predict patient response to chemoradiotherapy, whereby responses in patients matched patient-derived organoid responses [262,278,279,280].

The reverse genetic approached can be also used in organoid cultures to understand the relationship between a gene defect and disease phenotype, using conventional gene expression and knockout assays are used for this purpose. Interestingly, clustered regularly interspaced short palindromic repeats (CRISPR)–CRISPR-associated protein (Cas9)- is a novel genome editing tool that has been used in many model systems including organoids, to study the genotype-phenotype interaction in many diseases such as monogenic diseases and cancer [281]. CRISPR-Cas9 was initially used to reconstitute colorectal cancer in human organoids, using the classical genetic model of colorectal cancer progression. The most known editing in this regard is disruption of tumor suppressor genes, such as *p53* and *APC*, and introduction of oncogenic mutations (*KRAS* and *PIK3CA*) [282,283]. These mutations and others accelerated the acquisition of mutations that favor epithelial-to-mesenchymal transition and incremented tumorigenic capacity of initially normal colonic organoids [284]. This approach was implemented in other human normal organoids to replicate human gastric [257], pancreatic [267], liver [285], and breast cancer [286].

Organoid co-culture systems are being used to study the role of cancer-associated fibroblasts (CAFs), important component of tumor microenvironment. A recent study demonstrated that these cells could activate inflammation and promote carcinogenesis and tumor growth when co-cultured with mouse pancreatic cancer organoids [287]. It was also shown that pancreatic CAFs secrete Wnt ligands and support the growth of pancreatic tumor organoids through physical contact, specifically via juxtacrine molecular communication [267].

Co-culturing immune cells with organoids allowed for the study of interaction between epithelial and immune cells. Immunotherapy using chimeric antigen receptor (CAR)-engineered lymphocytes was tested using patient-derived COs [288]. Results showed that CAR-engineered natural killer (NK-92) cells directed towards either epithelial cell adhesion molecule (EPCAM), epidermal growth factor receptor variant III (EGFRvIII), or FRIZZLED receptors demonstrated efficient targeting and sensitizing of CRC organoids to cell death. However, the targeting of FRIZZLED receptors induced cytotoxicity against epithelial cells in normal organoids [288]. Hybrid culture organoids with peripheral blood lymphocytes were used to enrich tumor-reactive T cells from peripheral blood of patients with colorectal and non-small-lung cancer. This study showed that enriched population can be used in a personalized manner to kill matched tumor organoids [10]. Air-liquid interface culture of human patient-derived tumor organoids was also implemented and showed conservation of original tumor T cell receptor spectrum [289]. Collectively, these studies show that co-culturing organoids with non-epithelial cells is a good strategy that allows the study of the complicated interaction between tumor and stromal cells.

#### 4.2.2. ASC-Derived Organoids and Cystic Fibrosis (CF)

Rectal organoids derived from patients with CF have been used to study and characterize personalized responses to CFTR-modulating drugs. Researchers found that the efficacy of these drugs depends on CFTR mutation and function and genetic background of patients [290]. Another study found that organoid response to these drugs correlates with clinical response parameters such as percentage of predicted forced expiratory volume in 1 s (ppFEV1) [291]. Findings from these studies suggest that patient-derived rectal organoids enable prediction of clinical response and personalized treatment of the disease.

#### 4.2.3. ASC-Derived Organoids and Infectious Diseases

Clinically relevant applications of organoids include the study infectious diseases with high inter-individual variations, due to several inherited genetic factors and age and gender differences. The growing and culturing of viruses is now possible by the establishment of human organoids. Human model systems are preferable to animal models, particularly when studying pathogenesis of viruses and other infectious diseases. This is because some pathogens are ecotropic, meaning they infect a narrow range of species or host tissues [39]. Before the establishment of human organoids, it was not possible to culture highly pathogenic viruses, such as the norovirus, the leading cause of acute gastroenteritis, whose pathogenic mechanisms were still unclear [292]. The establishment of intestinal organoids-derived epithelial monolayer allowed for the study of the host-virus interaction and evaluation of methods to prevent and treat infection. Findings from a study that used these models revealed that specific viral strains require bile components to infect intestinal epithelial cells [120]. Yin et al. successfully established human and mouse intestinal organoids, which were then used to cultivate experimental and patient-derived rotavirus trains. Treatment with ribavirin resulted in reduced rotavirus RNA levels in the established intestinal organoids [293]. Other viruses were also studied using human airway organoid model systems and include influenza virus [294,295,296], enteroviruses [297], human astrovirus [298], human adenovirus [299], human papillomavirus [300], BK virus [301], herpes simplex virus [300], and respiratory syncytial virus [274].

With the recent spread of COVID-19 pandemic, extensive efforts have been made to model and understand the biology and pathogenesis of severe acute respiratory syndrome coronavirus 2 (SARS-CoV-2) infection.Using adult-stem cell derived organoid models, several studies reported that SARS-Cov-2 can infect enterocytes, further confirming that the intestine is susceptible to infection [302,303]. Lamers et al. [302] demonstrated that viral replication in enterocytes resulted in viral response genes upregulation and the release of infectious viral particles [302]. A study also showed that viral entry into and infection of enterocytes in human small intestinal enteroids is mediated by two mucosa-specific serine proteases, TMPRSS2 and TMPRSS4. However, virus particles released into intestinal lumen were inactivated and therefore, infectious viruses were not detected in stool of specimens of COVID-19 patients [303]. Zhou et al. [304] established and characterized intestinal organoids derived from horseshoe bats of the Rhinolophus sinicus species and demonstrated active viral replication within these organoids. Furthermore, active viral replication was also demonstrated in human intestinal organoids [304]. In addition, the potential neurotoxic effect of SARS-CoV-19 is being studied using brain organoids. Ramani et al. [305] demonstrated that SARS-CoV-2 targets neurons of human brain organoids and alters distribution and phosphorylation of Tau, resulting in neuronal death [305]. Moreover, lung and colonic organoids allowed for the identification of SARS-CoV-2 inhibitors, namely imatinib, mycophenolic acid and quinacrine dihydrochloride [306].

Modeling bacterial and parasite infection is now possible using these systems. *Cryptosporidium*, a protozoan parasite, has been shown to infect intestinal and lung organoids derived from healthy tissues. The parasite was able to replicate and complete its entire life cycle within the organoids [307]. *Toxoplasma Gondii* is one of the most common parasitic infections in the general population and that specifically poses a risk to pregnant women, who might catch the infection from cats. A recent study established cat and mouse intestinal organoids and identified that the buildup of linoleic acid was due to the absence of delta-6-desaturase activity and is required for the sexual development of the parasite [308]. Another study established organoids derived from bovine and porcine intestinal crypts and infected them with Toxoplasma gondii [309].

Human intestinal organoids have been used to study the host-pathogen interaction, including *C. difficile* infection, the leading cause of infectious nosocomial diarrhea [212]. Human enteroids, epithelial spheroids derived from gastrointestinal tissue, are a promising model for studying host-pathogen interaction. However, a major limitation is accessing the apical enteroid surface within the organoids. Co et al. [310] devised a model system with reverted polarity to overcome this issue. The novel model exhibited proper polarity and barrier function. This model enabled the study of the mode of entry of *Salmonella enterica serovar Typhimurium* and *Listeria monocytogenes* that exploit cell polarity of epithelial cells [310].

Subsequently, similar approaches were followed to infect human organoids with a wide range of bacteria and parasites, and study host-pathogen interactions. Examples of bacteria studied using human gastric and intestinal organoids include *Helicobacter pylori* [140,311,312], *Salmonella typhimurium* [309,310,313], *Escherichia coli* [314,315,316], *chlamydia trachomatis* [317], *C. difficile* [308], and *Klebsiella pneumoniae* [318].

ASC-derived organoid models have presented significant results within the modeling of human disease for a broad spectrum of life stages, early development and throughout to adulthood.

It is worth noting that there is a difference in ASC-derived organoid cultures between mouse and human systems. This has signified the need for human-based laboratory model systems to fully understand human development and pathophysiology.

Based on the source of cells, Table 1 and Table 2 highlight the differences in generation methods and recapitulation of the native organ’s physiology respectively.

## 5. Safety Concerns

3D organoid technology will potentially revolutionize the way medicine functions whether by studying disease intricacies, testing dugs, or exploring specific patient-oriented applications [319]. But as with anything new and not completely elucidated, organoids research embodies several safety concerns. One such safety issue arises from the fact that the iPSCs, from which organoids are derived, might lack genetic stability. Because these precursor cells remain in culture for prolonged periods of time, they are prone to accumulate genetic modifications such as copy number variants and chromosomal aberrations. Optimization of reprogramming methodologies and culturing conditions may help decrease the aforementioned genetic instabilities, or at least allow for better control of downstream outcomes. [320]. It is noteworthy that in the first clinical trial involving iPSCs for age-related macular degeneration, the second patient, out of a total of two, was not treated due to fears of the possible effects of the DNA copy number deletions detected, particularly that one of those deletions lies on an X chromosome and the patient was male [321]. Kim et al. showed that in vitro cellular expansion of mesenchymal stromal cells leads to somatic mutation accumulation via certain mutational bursts. Seeing as these cells form the scaffold upon which organoid culturing depends on, the mutations detected by whole genome sequencing need to be considered in terms of clinical applications, particularly cell-based therapeutic approaches which require this step of ex vivo expansion. Delving into the proliferation history may allow us to predict genomic instability. However, it goes without saying that further investigation is necessary since extrapolation from a small cohort size warrants caution [322]. The extracellular matrix also affects the in vitro growth of cells. The reconstituted basement membrane derived from Engelbreth-Holm- Swarm, Matrigel, is often used to boost the rate of engraftment in PDX models but which can be the origin of many murine viruses, contaminating the sample [323]. A novel strategy that may be explored to mitigate the risks of stem cell mutations is suicide gene therapy. The two main avenues of suicide therapy are cytosine deaminase/5-fluorocytosine and HSV/ganciclovir which would potentially serve to kill the aberrant cells with accumulated mutations; an important point to tackle in regenerative medicine and prior to any potential transplantation [324]. Another safety concern that arises which also has implications on the effectiveness of these organoid systems is that when the cultured cells amass mutations, they become less representative of how their counterparts would have behaved in vivo. As such, toxicological testing conducted in some cancer cell lines for example may not produce reliable and consistent screenings, undermining the value of translating these results into clinical practice. One possible solution is to generate iPSCs via methods other than DNA recombination such as using adenoviral vectors and plasmids [325]. Finally, Yamanaka addressed two other major challenges in pluripotent stem-cell based therapies: tumorigenicity and immunogenicity. The teratogenic potential may be restricted by either employing more stringent purification methods that decrease the number of residual PSCs or by limiting the use of highly tumorigenic reprogramming factors (such as c-myc) when generating iPSCs. Immune rejection that is especially seen in allografts could be restrained by using immunosuppressants as with classical organ transplantation. Matching of HLA haplotypes and employing novel gene editing based techniques such as HLA cloaking (whereby HLA genes are inactivated) can also help in minimizing organoid graft rejection [326].

## 6. Conclusions and Future Directions

**Figure 3:**
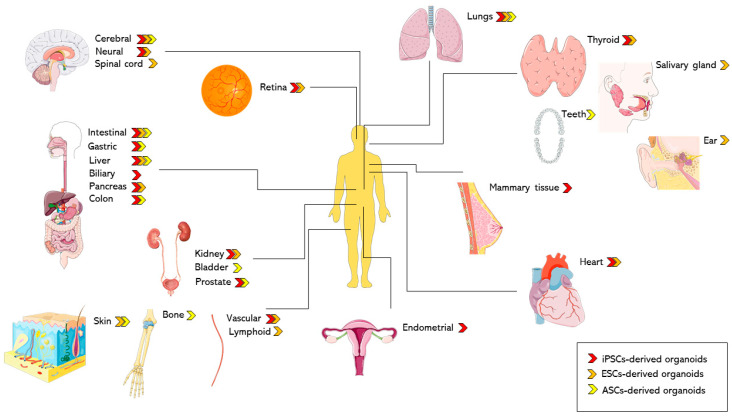
Reported organoid systems based on tissue of origin. Using different types of stem cells, many organs and tissues have been simulated using the organoids technology. Organ and human model images are imported from Servier Medical Art by Servier (http://smart.servier.com/), accessed on 24 May 2021. Licensed under a Creative Commons Attribution 3.0 Unported License (https://creativecommons.org/licenses/by/3.0/).

In conclusion, although there might be many challenges hindering the use of organoids for clinical applications, this new technology holds great potential in translational research. Organoid technology has recently grown to incorporate drug screening, disease modeling, genetic manipulation, omics analysis, and many others (Figure 2). Experimental procedures that have been developed to culture organoids can be applied to different human organ systems, helping us better understand the human biology and development of diseases (Figure 3, Table 3). Indeed, human organoid systems are strongly proposed to offer exceptional opportunities to improve human health and move the biomedical and research fields to a whole new level of personalized patient-targeted medicine.

## Figures and Tables

**Table 1:** Differences in generation methods of several organoid models based on the source of cells.

Organ Model	iPSC-Derived Organoids	ESC-Derived Organoids	ASC-Derived Organoids
Intestinal	- Human-PSC-derived NCCs and developing human intestinal organoids were combined in vitro [43] - Patient-specific iPSCs harboring an LRRK2 G2019S mutation (LK2GS) [98] - Reprogramed fibroblasts from normal and FAP patients using the STEMCCA system [110] - A1ATD-1 hIPSCs derived from skin fibroblasts [122]	- Endoderm induction and differentiation into intestinal organoids [42]	- Co-culture of tumor cells and stromal cells supporting cancer development. Intestinal crypts and FAC sorted Lgr5+ intestinal epithelium cells embedded in Matrigel - Mouse- and human-derived colonic stem cells [230]
Lung	- hiPSCs [53,54,57]	- Endoderm induction and differentiation of hESCs to lung progenitor cells, 3D lung organoid formation, branching, and maturation [164]	- Lung epithelial cells sorted from human bronchi (excess unaffected areas). These cells had self-renewal and could generate luminal daughters [249] - Five subtypes (adenocarcinoma, squamous cell carcinoma, small cell carcinoma, adenosquamous carcinoma, and large cell carcinoma) of cancer tissues to generate human lung cancer organoids (LCOs) [273]
Cerebral	- Human iPSCs-derived embryoid bodies generated neuroectoderm which gave rise to cerebral tissues [89] - Miller-Dieker Syndrome (MDS) normal and patient-derived hiPSCs [94]	- Embryoid bodies formation, neural induction [175,176]	- Patient-derived glioblastoma organoids [263] - Patient-derived organoids from metastatic neuroblasoma patients [264]
Liver	- hiPSCs [47,48,49]	- Human expandable hepatic organoids (hEHOs) from hESCs with totally defined (serum-free, feeder free) media. 3D co-cultures of hepatic organoids were generated with hEHOs and human fetal liver mesenchymal cells to recapitulate the aspects of the human alcoholic liver disease-associated pathophysiology after ethanol treatment [171].	- Tumor organoids from patients who had primary liver cancer [268]

**Table 2:** Differences in the recapitulation of the native organ’s physiology based on the source of cells.

Organ Model	iPSC-Derived Organoids	ESC-Derived Organoids	ASC-derived Organoids
Intestinal	- Enteric nervous system development, neuroglial structures, functional interstitial cells of Cajal [43] - Spheroid structures with a central empty lumen and some crypt-like structures comprised all intestinal epithelial cell types [98] - Gut organoids exhibiting definitive endoderm and intestinal specification. Differences in the generated intestinal-like tissues from normal or heterozygous APC iPSC were linked to the inter-patient variability [110] - Crypt-like structures, polarized epithelial cells, microvilli on the apical surface, enterocyte interspersed with Paneth, goblet, and enteroendocrine cells [122]	- Polarized, columnar epithelium that was patterned into villus-like structures and crypt-like proliferative zones that expressed intestinal stem cell markers. The epithelium contained functional enterocytes, as well as goblet, Paneth, and enteroendocrine cells [42]	- Exhibition of an adult phenotype and recapitulation of the in vivo tissue architecture [230].
Lung	- Mesenchymal and lung epithelial cells along with upper airway-like structures with basal cells, ciliated cells, and club cells surrounded by smooth muscle and myofibroblasts in addition to a distal-airway-like structures with bipotent alveolar progenitor cells [53] - Mesoderm and pulmonary endoderm, developed into branching airway and early alveolar structures and yielded growths containing tubular structures surrounded by mesenchymal tissue [54] - Synchronized modulation of proximal airway versus distal alveolar epithelial patterning [57]	- Multiple cell types such as epithelial, mesenchymal, as well as ciliated cells. Organoids mimic the development and the function of a mature lung [164].	- Authors did not mention if they resembled the original epithelium, but they definitely do not resemble the organ’s developmental physiology nor complexity because they lack other cells, such as stroma or columnar epithelial cells [249] - LCOs maintained the histology, and marker (CK7 and p63) expression pattern, and genetic characteristics (ex. Mutations in TP53 and EGFR) of the original tumor they were derived from [273]
Cerebral	- Discrete brain regions including forebrain,- regional subspecification of cortical lobes and other regions such as the hippocampus, ventral forebrain, choroid plexus and immature retina [89] - Recapitulated MDS pathogenesis with horizontal cleavage planes in the ventricular zone, decreased vertical divisions, increased apoptosis of neuroepithelial stem cells, defective neuronal migration and abundance of CTIP2-positive neurons [94]	- Features of the human midbrain, but not the forebrain or the hindbrain [175] - Forebrain organoids which contain mainly dorsal forebrain neurons. Markers of midbrain and hindbrain were not observed [176]	- Organoids maintained biological features of high-grade glioblastoma (H&E staining), presence of the hall mark of glioblastoma, hypoxia and micro-vasculature (confocal imaging). Immunohistochemical analysis also showed the expression of neural progenitor and glioma stem cell markers. They also maintained molecular and intra- and inter-tumor heterogeneity [263] - The established organoids recapitulated the histological characteristics of the tumor (shown through H&E staining and immunohistochemical staining of NB84 (neuroblastoma diagnostic marker). The organoids also exhibited the disease-specific chromosomal aberrations, stemness properties, and tumor heterogeneity [264]
Liver	- Epithelialized cystic and/or ductal structures that express mature bile ducts markers including the CFTR marker, exhibit cystic and/or ductal structures and epithelial functions including CFTR-mediated fluid secretion [47] - Cholanginocyte progenitors formed cystic organoids and branching tubular structures having primary cilia and expressing biliary markers similar to primary chlangiocyte, Cholangiocyte-like cells possessed functional activity including bile acids transfer, alkaline phosphatase activity, γ-glutamyltranspeptidase activity and physiological responses to secretin, somatostatin and vascular endothelial growth factor [48] - Vascularized and functional human liver. The generated liver tissue is capable of protein production and human-specific drug metabolism [49]	- hEHOs exhibit the phenotype of hepatic stem/progenitor cells. The cells from hEHOs shows exceptional repopulation capacity in injured livers of FRG mice after transplantation, and they can differentiate in vivo into both mature hepatocytes and cholangiocytes [171]	- Hepatocellular carcinoma tumoroids had compact structures, however, cholangiocarcinoma tumoroids had irregular cyst structure thus recapitulating native physiology [268]

**Table 3:** Table representing the multiple organ and disease models based on their respective organoid origin (iPSC, ESC and ASC).

	iPSC-Derived Organoids	ESC-Derived Organoids	ASC-Derived Organoids
Organ models	▪ Intestinal [42,43,98,120,121,122,304] ▪ Gastric [41] ▪ Biliary [47] ▪ Liver [47,48,49] ▪ Thyroid [52] ▪ Lung [53,54,57,59] ▪ Kidney [62,63,67] ▪ Cardiac [68,69,70,72,73] ▪ Vascular [85] ▪ Female reproductive tract [86] ▪ Endometrial [87,88] ▪ Cerebral [89,94,97,113] ▪ Neural [90,96,98] ▪ Ventral and dorsal forebrain [91,95] ▪ Neuromuscular junctions [99,100,101] ▪ Mammary tissue [102,103] ▪ Retina [104,105,106,107] ▪ Colon [110,111] ▪ Pancreas [112]	▪ Lung [164] ▪ Pancreas [165,166,167] ▪ Liver [171] ▪ Prostate [173] ▪ Midbrain [175] ▪ Brain cortex [176] ▪ Vascular [177] ▪ Lymphoid [178] ▪ Kidney [62,180] ▪ Thyroid [52,181] ▪ Heart [183] ▪ Ear [187,188] ▪ Salivary gland [190] ▪ Skin [191] ▪ Retina [195,209] ▪ Spinal cord [196,201] ▪ Motor neurons [197,198] ▪ Forebrain [199] ▪ Intestinal [212]	▪ Prostate [8] ▪ Intestinal [230,231] ▪ Colon [233] ▪ Stomach [232,233] ▪ Liver [234] ▪ Brain [39] ▪ Bone [243] ▪ Lungs [249,250] ▪ Teeth [253] ▪ Salivary gland [254]
Disease models	▪ Hirschsprung’s disease [43] ▪ *Helicobacter pylori* infection [41] ▪ Alagille syndrome [48] ▪ Cystic fibrosis [47,48,59,238] ▪ Polycystic kidney disease [48,62,66] ▪ Barth syndrome [72] ▪ Genetic Cardiomyopathy [73] ▪ Cardiac Injury [68] ▪ Diabetic Vasculopathy [85] ▪ Endometrial disorders [88] ▪ Microcephaly [89] ▪ Autism spectrum disorder [90] ▪ Timothy syndrome [91] ▪ Tuberous sclerosis [93] ▪ Lissencephaly [94] ▪ Miller-Dieker syndrome [95] ▪ Alzheimer’s disease [96] ▪ Huntington’s disease [97] ▪ Parkinson’s disease [98] ▪ Retinal ciliopathies [104] ▪ X-linked juvenile retinoschisis [105] ▪ Late-onset retinitis pigmentosa [106,107] ▪ Li-Fraumeni syndrome- associated osteosarcoma [108] ▪ Colorectal cancer [110,111] ▪ Pancreatic adenocarcinoma [112] ▪ Brain tumors [113] ▪ Middle East Respiratory Syndrome-related Coronavirus infection [119] ▪ Norovirus infection [120] ▪ Rotavirus infection [121] ▪ *Salmonella enterica serovar Typhimurium* infection [122] ▪ Herpes simplex virus 1 encephalitis [125] ▪ Respiratory syncytial virus infection [54]	▪ SARS-CoV-2 infection [164] ▪ Diabetes [168,194] ▪ Steatohepatitis [171,172] ▪ Parkinson’s disease [175] ▪ Polycystic kidney disease [62] ▪ Hypothyroidism [181] ▪ Myocardial infraction [184,185,186] ▪ Lesch-Nyhan syndrome [193] ▪ Retinoblastoma [209] ▪ Metastatic brain cancer [210] ▪ *Clostridium difficile* infection [212] ▪ ZIKV infection and microcephaly [213]	▪ ZIKV infection and microcephaly [213,240,242] ▪ Gastric cancer [255,256,257] ▪ Colorectal cancer [258,259,260,261,262,284] ▪ Brain cancer [263,264] ▪ Pancreatic cancer [265,266,267,287] ▪ Liver cancer [268,285] ▪ Bladder cancer [269] ▪ Prostate cancer [270] ▪ Breast cancer [271,286] ▪ Esophagus cancer [272] ▪ Lung cancer [273,274] ▪ Endometrial cancer [275] ▪ Cystic fibrosis [290,291] ▪ Rotavirus infection [293] ▪ Influenza virus infection [294,295,296] ▪ Enteroviruses infection [297] ▪ Human astrovirus infection [298] ▪ Human adeno-virus infection [299] ▪ Human papillomavirus infection [300] ▪ BK virus infection [301] ▪ Herpes simplex virus infection [300] ▪ Respiratory syncytial virus infection [274] ▪ SARS-Cov-2 infection [302,303,304,305,306] ▪ *Cryptosporidium* infection [307] ▪ *Toxoplasma Gondii* infection [308,309] ▪ *Clostridium difficile* infection [212] ▪ *Salmonella enterica serovar Typhimurium* infection [309,310,313] ▪ *Listeria monocytogenes* infection [310] ▪ *Helicobacter pylori* infection [140,311,312] ▪ *Escherichia coli* infection [314,315,316] ▪ *Chlamydia trachomatis* infection [317] ▪ *Klebsiella pneumoniae* infection [318]

## Data Availability

Not applicable.
